# Redo cardiac surgery for active prosthetic valve endocarditis associated with hereditary hemorrhagic telangiectasia: report of a case

**DOI:** 10.1007/s00595-014-0876-6

**Published:** 2014-03-20

**Authors:** Yuki Nakamura, Fumiaki Shikata, Masahiro Ryugo, Toru Okamura, Takumi Yasugi, Hironori Izutani

**Affiliations:** Department of Cardiovascular Surgery, Ehime University Hospital, Shitsukawa, Toon, Ehime 791-0295 Japan

**Keywords:** Hereditary hemorrhagic telangiectasia, Prosthetic valve endocarditis, Cardiac surgery

## Abstract

Hereditary hemorrhagic telangiectasia (HHT) is caused by an autosomal dominant gene and characterized by multiple arteriovenous malformations in several organs, leading to bleeding or shunting. These patients often suffer severe infections and heart failure, which should be managed in the perioperative period, when open heart surgery is indicated. We report a case of successful aortic root replacement for active prosthetic valve endocarditis and ventricular septal perforation in a patient with HHT, who had severe heart failure.

## Introduction

Hereditary hemorrhagic telangiectasia (HHT), an autosomal dominant genetic disorder, is characterized by multiple organ involvements such as cutaneous or mucosal telangiectasia and visceral multiple arteriovenous malformations (AVMs), which develop in the brain, liver, or lungs. Abnormal vessel formation and subsequent bleeding form the basis of most clinical manifestations such as recurrent epistaxis. Bleeding, shunting, thrombosis and embolus frequently occur as complications of AVMs [1]. Although the AVMs often cause sudden and catastrophic infections and heart failure, the mechanisms underlying these phenomena remain unknown. There are few reports on the surgical treatment of active infectious endocarditis in HHT. Thus, we describe a successful operation for active prosthetic valve endocarditis (PVE) and ventricular septal perforation in a patient with HHT who had severe heart failure.

## Case report

The patient was a 65-year-old woman, who had undergone aortic valve replacement for severe aortic stenosis with a bicuspid valve, using a bioprosthesis (19-mm Carpenter-Edwards Perimount valve, Edwards Lifesciences, Irvine, CA, USA) 10 years earlier, at our institution. At that time, HHT was diagnosed and treated with combined ethinyl estradiol and norgestrel therapy, as reported previously [2].

**Fig. 1:**
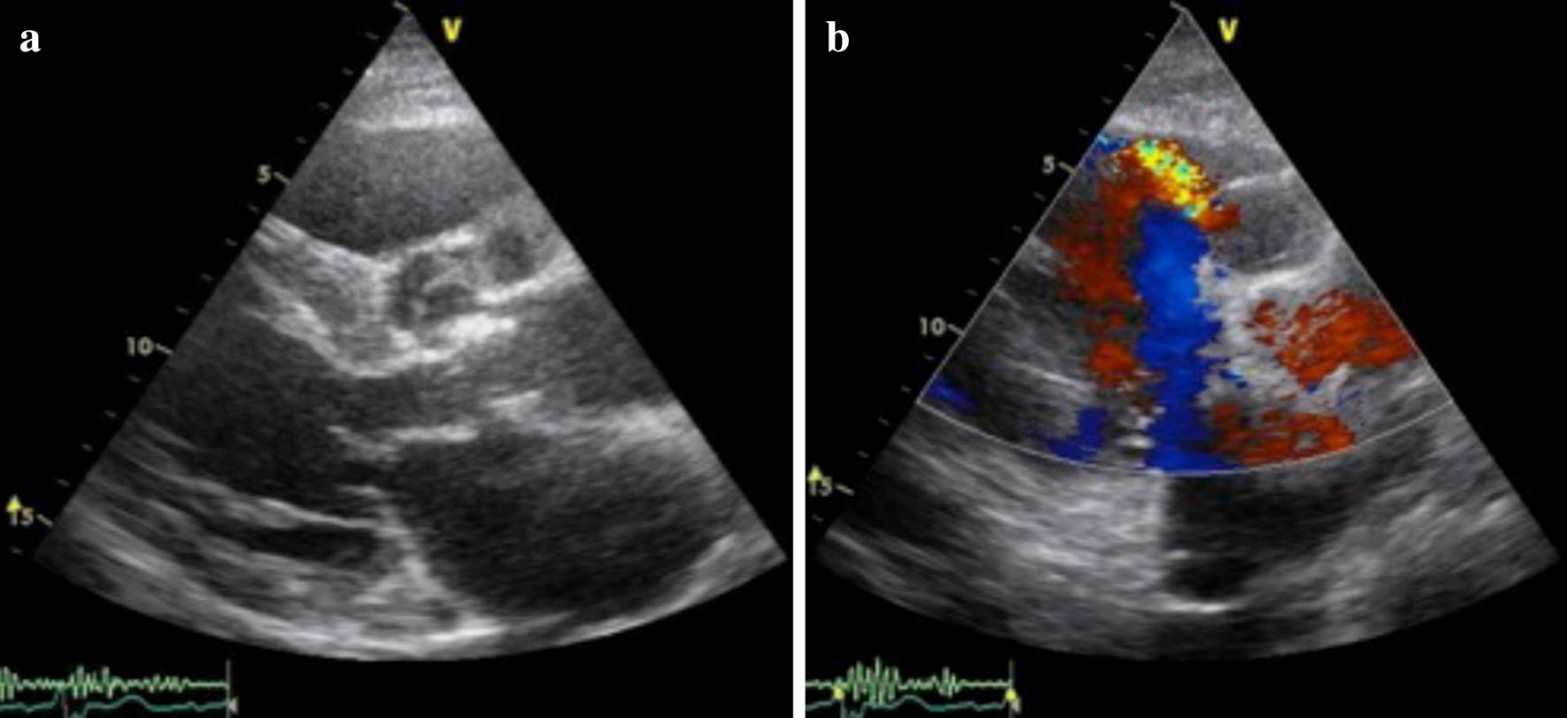
**a** Transthoracic echocardiogram showing vegetation on the bioprosthesis and annular abscess; **b** transthoracic echocardiogram showing shunt flow from the left to the right ventricle

**Fig. 2:**
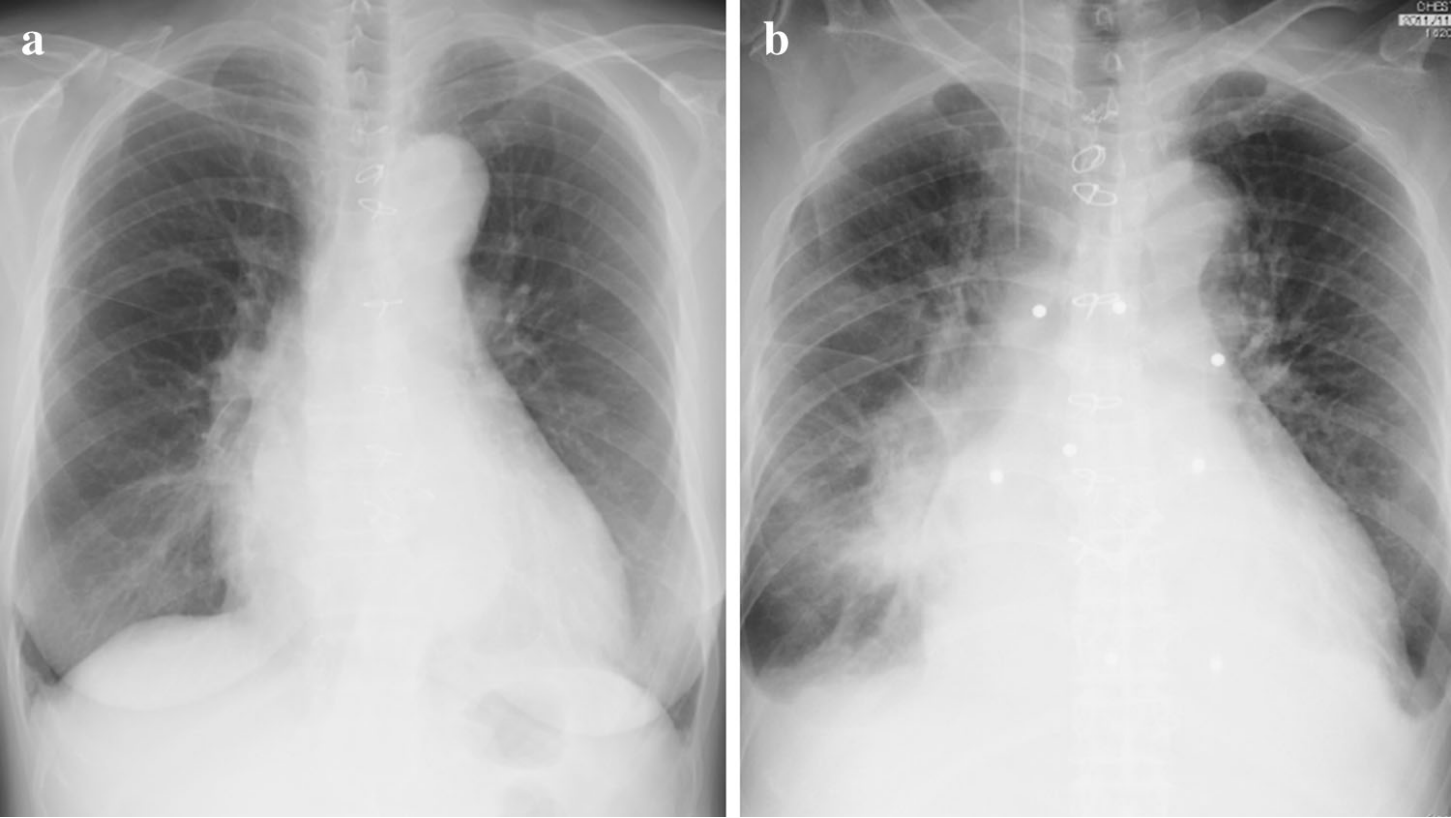
**a** Chest radiography on admission. **b** Chest radiography just before surgery

**Fig. 3:**
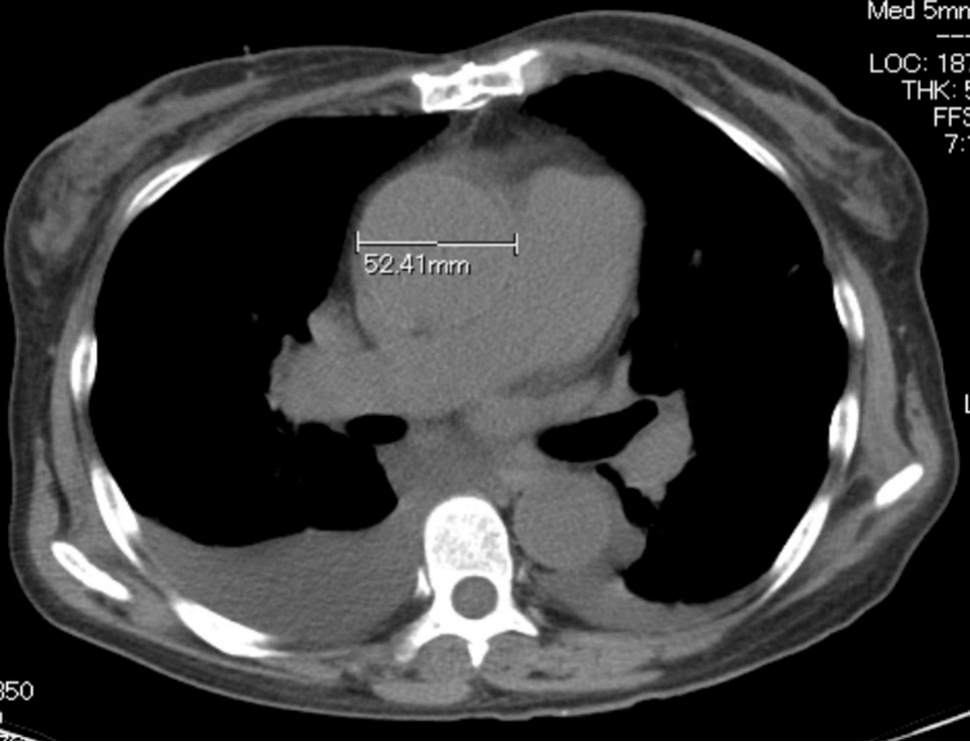
Preoperative computed tomography showed a dilated ascending aorta

About 1 year prior to the present admission for the fever and recurrent epistaxis, nasal mucosal telangiectasia had appeared, followed by the increased frequency of epistaxis. Blood cultures indicated the presence of methicillin-sensitive *Staphylococcus epidermidis* and echocardiogram showed prosthetic valve dysfunction with aortic regurgitation and vegetation. Despite intravenous antibiotics, congestive heart failure ensued. We administered diuretics and inotropes to maintain a satisfactory blood pressure and she was transferred to the intensive care unit (ICU) to improve her general condition. Her second echocardiogram showed evidence of ventricular septal perforation, a swaying prosthetic valve (Fig. 1a, b), and severe tricuspid regurgitation. The pressure gradient across the tricuspid valve was 54 mmHg. Chest radiography suggested congestive heart failure (Fig. 2). On preoperative computed tomography, the diameter of the ascending aorta was 52 mm (Fig. 3). Her progressive congestive heart failure and uncontrollable PVE warranted emergency surgery. Before the surgery, her condition stabilized without the need for intra-aortic balloon pumping (IABP) and tracheal intubation.

**Fig. 4:**
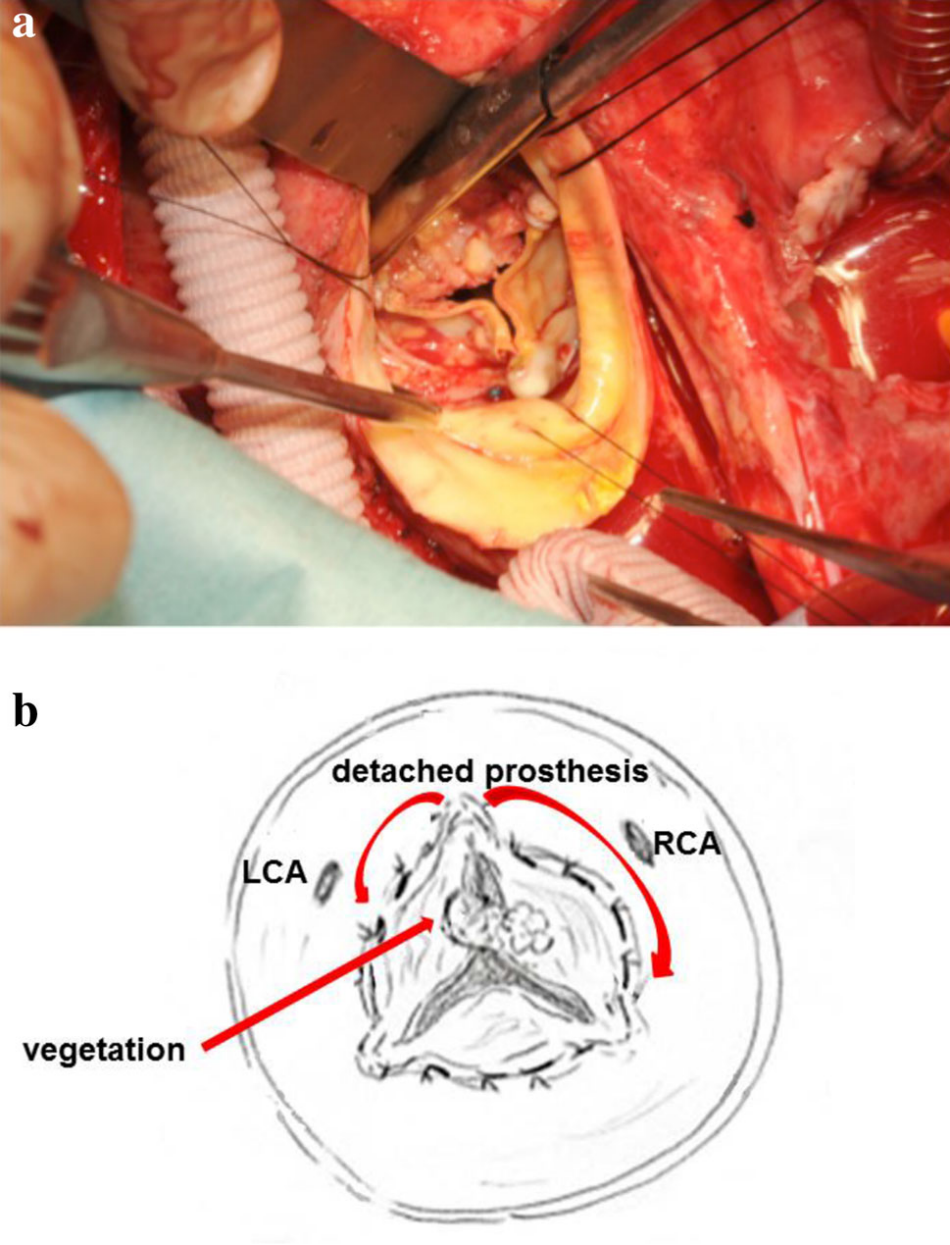
**a** Vegetation on the leaflet of the degenerated bioprosthesis. The prosthesis was partially detached from the annulus. **b** *Right arrow* in the figure shows the range of detachment of the prosthesis from the annulus. *RCA* right coronary artery, *LCA* left coronary artery

**Fig. 5:**
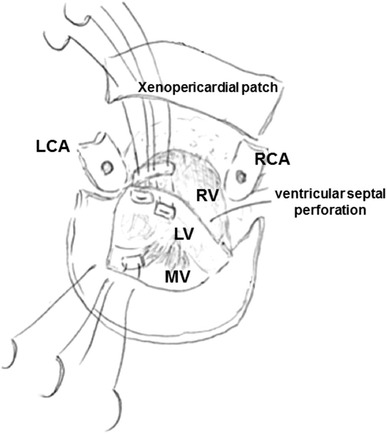
This operative schema shows how we repaired the ventricular septal perforation. *RCA* right coronary artery, *LCA* left coronary artery, *RV* right ventricle, *LV* left ventricle, *MV* mitral valve

After inserting a tracheal tube carefully, but not a nasogastric tube, median sternotomy was performed. Systemic heparinization was delivered, and the patient’s activated clotting time was maintained at above 400 s throughout the surgery. Her primary otolaryngologist had waited until the operation was finalized to observe her nasal condition and stop any bleeding. An aortic cannula was inserted from the ascending aorta and cardiopulmonary bypass was established with bicaval cannulation. The ascending aorta was opened and trimmed just below the brachiocephalic artery under moderate hypothermic circulation arrest (28 °C) and retrograde cerebral perfusion. A 24-mm tube graft (GelweaveDacron graft, Vascutek USA, Inc., Ann Arbor, MI) with a side branch was anastomosed to the distal ascending aorta. We observed vegetation on the leaflet of the degenerated bioprosthesis. The prosthesis was partially detached from the annulus (Fig. 4). There was ventricular septal perforation below the valve along the muscular and membranous septum and the annulus, which was severely damaged by the endocarditis. The fragile tissue was removed carefully, following which the damaged annulus was reconstructed, and the perforated septum was repaired with a xenopericardial patch. A 27-mm Freestyle aortic root bioprosthesis (Medtronic Inc., Minneapolis, MN, USA) was placed in the supra-annular position and the left and right coronary arteries were reconstructed. Finally, the proximal end of the graft was anastomosed to the distal end of the bioprosthesis (Fig. 5).

While in ICU, the patient suffered low cardiac output syndrome and IABP was initiated to stabilize the blood pressure. A few days after the operation, copious serous secretions started oozing from the trachea. We observed a similarity in the constituents of this secretion and those of her plasma, hence she was given a transfusion of fresh frozen plasma and administered a steroid. Coagulated serous secretions were removed from the inferior part of the right lung via bronchoscopy. The antibiotic infusion was continued for more than 6 weeks postoperatively, but she was not given anticoagulants or antiplatelet drugs. The postoperative echocardiogram showed no evidence of recurrence of the infective endocarditis (IE) or heart failure. Her recurrent epistaxis also improved. When last seen, 9 months after her operation, the patient was in good health, without remarkable symptoms.

## Discussion

The cutaneous or mucosal telangiectasia and AVM associated with HHT can cause multiple complications, which are difficult to manage, and may result in life-threatening events perioperatively. Several cases of open heart surgery in patients with HHT have been reported; however, active hemorrhage caused by cutaneous or mucosal telangiectasia in the preoperative period is rare. Although Sakata et al. [2] suggested various methods for preventing abnormal postoperative hemorrhage, its management remains unclear. In the present report, we described successful redo cardiac surgery in a patient with HHT, managed perioperatively, in addition to a rare complication likely associated with HHT.

Because of the AVMs, infections in HHT patients occur primarily in the lungs, brain, and liver; however, IE and especially PVE, rarely occur with HHT. Tooth decay, atopic dermatitis, and other infectious diseases have been described as causes of IE [3]; however, this patient did not have any of these disorders. Considering previous case reports of endocarditis caused by nasal packing [4, 5], we suspect that the PVE in our patient resulted from her anterior nasal packing for recurrent epistaxis but that bacteria were not trapped because of the pulmonary AVM. As recurrent epistaxis may increase the risk of IE, her persistent nasal telangiectasia requires long-term follow-up.

There are a few reports of complications such as high cardiac output failure, shunt cyanosis due to pulmonary AVM, and bleeding from mucosal telangiectasia, occurring perioperatively in HHT patients undergoing cardiac surgery for IE. Ishikawa et al. [6] suggested that surgery may be inappropriate for an HHT patient with AVM or mucosal telangiectasia because of the risk of life-threatening bleeding. Although we knew that our patient had AVMs and mucosal telangiectasia before her operation, she suffered only mild hemorrhage perioperatively. Her recurrent epistaxis resolved after the operation, as described in our previous case report [7]. Several measures were taken during the preoperative and postoperative periods to prevent life-threatening hemorrhage, including the administration of estrogen before the operation, not inserting a nasogastric tube, and discontinuance of anticoagulant drugs pre- and postoperatively [8, 9]. Thus, we believe that cardiac surgery should be performed for cardiac failure in patients with HHT, even if they have recurrent hemorrhages from mucosal telangiectasia. We also suggest that capillary syndrome is a perioperative complication of AVM in patients with HHT. Pulmonary AVMs in HHT occur most frequently in the inferior lobe of the lungs [1], the vasopermeability of which is enhanced by stress such as inflammation after cardiopulmonary bypass. In the present case, we suspect that the capillary syndrome triggered the serous secretion overflow into the trachea from the inferior lobe.

In summary, we reported a successful operation for progressive PVE in a patient with HHT. HHT patients undergoing open heart surgery are vulnerable to various perioperative risks and complications which can be life-threatening. Nonetheless, we recommend performing open heart surgery for these patients, with proper management in the perioperative period.
